# Complete Genome Sequences of Three Representative *Mycobacterium tuberculosis* Beijing Family Strains Belonging to Distinct Genotype Clusters in Hanoi, Vietnam, during 2007 to 2009

**DOI:** 10.1128/genomeA.00510-17

**Published:** 2017-07-06

**Authors:** Takayuki Wada, Minako Hijikata, Shinji Maeda, Nguyen Thi Le Hang, Pham Huu Thuong, Nguyen Phuong Hoang, Nguyen Van Hung, Naoto Keicho

**Affiliations:** a Department of International Health, Institute of Tropical Medicine, Nagasaki University, Nagasaki, Japan; b Department of Pathophysiology and Host Defense, Research Institute of Tuberculosis JATA, Tokyo, Japan; c Hokkaido Pharmaceutical University School of Pharmacy, Sapporo, Japan; d NCGM-BMH Medical Collaboration Center, Hanoi, Vietnam; e Hanoi Lung Hospital, Hanoi, Vietnam; f Department of Microbiology, Hanoi Lung Hospital, Hanoi, Vietnam; g Department of Microbiology, National Lung Hospital, Hanoi, Vietnam; h National Center for Global Health and Medicine, Tokyo, Japan

## Abstract

We present here three complete genome sequences of *Mycobacterium tuberculosis* Beijing family strains isolated in Hanoi, Vietnam. These three strains were selected from major genotypic clusters (15-MIRU-VNTR) identified in a previous population-based study. We emphasize their importance and potential as reference strains in this Asian region.

## GENOME ANNOUNCEMENT

Tuberculosis remains a major health threat worldwide, with 6.1 million new cases in 2015 and 49 million deaths between 2000 and 2015 (1). Of the seven major lineages of *Mycobacterium tuberculosis* in the world, Beijing family strains belong to lineage 2, the East-Asian lineage, and are endemic to the eastern part of Asia (2). This family has attracted the attention of many researchers because of its associated drug resistance, relapse, and transmissibility.

Vietnam has a high incidence of tuberculosis (1), with a high prevalence of Beijing family strains (3–5). In a previous population-based study in Hanoi, the capital of Vietnam, between 2007 and 2009 (4), we analyzed genotypic clusters defined by identical patterns of variable numbers of tandem repeat polymorphisms (15-MIRU-VNTR) to assess recent transmission (data not shown).

Here, we report the complete genome sequences of representative *M. tuberculosis* Beijing family strains (HN-205, HN-321, and HN-506) in the three major genotypic clusters. All strains were isolated from Vietnamese patients living in Hanoi (4). Phylogenetically, HN-205 and HN-506 belonged to the modern Beijing subfamily, ST10 (6, 7), whereas HN-321 belonged to the ancient Beijing subfamily, ST19/25.

Long-read sequences obtained using a PacBio RS II instrument (Pacific Biosciences, USA) were assembled with the Hierarchical Genome Assembly Process (HGAP) version 3. Consequently, a single contig was assembled in each of the strains. These contigs were polished by mapping analysis of 300-bp paired-end reads by MiSeq sequencing (Illumina, USA), using CLC Genomics Workbench version 7.5.2 (Qiagen, USA). After polishing, sequence regions with low coverage (<5) by remapping analysis were confirmed by Sanger sequencing.

The lengths of the complete genome sequences were 4,411,033 bp for HN-205, 4,421,540 bp for HN-321, and 4,413,362 bp for HN-506. G+C contents were all 65.6%. Prior to submission to DDBJ, D-FAST (8), a pipeline that includes annotation by Prokka version 1.11 (9), was applied, which predicted 4,059 genes for HN-205, 4,066 genes for HN-321, and 4,064 genes for HN-506, in addition to 52 tRNAs for all strains. The original annotation by D-FAST was modified to include locus tags and gene names of the H37Rv genome sequence (DDBJ no. AL123456.3), according to reciprocal BLASTp best hits by stand-alone BLAST+ version 2.2.29 (10).

Despite a lack of previous tuberculosis treatment history, conventional drug susceptibility tests for the first-line drugs reveled that HN-321 and HN-506 were both resistant to isoniazid (data not shown), with a *kat*G gene mutation at codon 315 (Ser to Thr). Streptomycin resistance was identified only in HN-321, with an *rps*L mutation at codon 43 (Lys to Arg). HN-205 was susceptible to all antibiotics.

As reference strains, the complete genome sequences reported here will be helpful in public health and in studies on transmission and drug resistance of Beijing strains in Vietnam.

### Accession number(s).

This whole-genome sequencing study has been deposited at DDBJ/ENA/GenBank under the accession numbers AP018034 (HN-205), AP018035 (HN-321), and AP018036 (HN-506). The versions described in this paper are the first versions.
